# *In vivo* single-RNA tracking shows that most tRNA diffuses freely in live bacteria

**DOI:** 10.1093/nar/gkw787

**Published:** 2016-09-12

**Authors:** Anne Plochowietz, Ian Farrell, Zeev Smilansky, Barry S. Cooperman, Achillefs N. Kapanidis

**Affiliations:** 1 Biological Physics Research Group, Clarendon Laboratory, Department of Physics, University of Oxford, Parks Road, OX1 3PU, Oxford, UK; 2 Anima Inc, 75 Claremont Road, Suite 102, Bernardsville, NJ 07924-2270, USA; 3 Department of Chemistry, University of Pennsylvania, 231 S. 34 Street, Philadelphia, PA 19104-6323, USA

## Abstract

Transfer RNA (tRNA) links messenger RNA nucleotide sequence with amino acid sequence during protein synthesis. Despite the importance of tRNA for translation, its subcellular distribution and diffusion properties in live cells are poorly understood. Here, we provide the first direct report on tRNA diffusion localization in live bacteria. We internalized tRNA labeled with organic fluorophores into live bacteria, applied single-molecule fluorescence imaging with single-particle tracking and localized and tracked single tRNA molecules over seconds. We observed two diffusive species: fast (with a diffusion coefficient of ∼8 μm^2^/s, consistent with free tRNA) and slow (consistent with tRNA bound to larger complexes). Our data indicate that a large fraction of internalized fluorescent tRNA (>70%) appears to diffuse freely in the bacterial cell. We also obtained the subcellular distribution of fast and slow diffusing tRNA molecules in multiple cells by normalizing for cell morphology. While fast diffusing tRNA is not excluded from the bacterial nucleoid, slow diffusing tRNA is localized to the cell periphery (showing a 30% enrichment versus a uniform distribution), similar to non-uniform localizations previously observed for mRNA and ribosomes.

## INTRODUCTION

Studying the subcellular distribution of RNA in living cells is vital for understanding the spatial organization of gene expression (1), since this distribution can control the processes of transcription and translation and efficiently alter protein activity. There are several examples of local RNA enrichment in both eukaryotic and prokaryotic cells (2). For instance, a preferential localization of *ASH1* mRNA encoding for an unstable transcriptional repressor protein in daughter cells has been shown in budding yeast (3), and translation-independent subcellular localization of transcripts encoding for membrane and soluble proteins has been observed in bacteria (4).

The subcellular RNA localization can be explored further through detection of individual RNA molecules and measurement of their heterogeneity, a challenge that can be met using single-molecule fluorescence microscopy. Initial studies to visualize single mRNA molecules in cells used fluorescence *in situ* hybridization (FISH) in fixed cells (5) and mRNA tracking in live cells (6). Subsequent *in vivo* studies of mRNA dynamics relied on indirect tagging of mRNA with fluorescent proteins (FP) fused to the bacteriophage MS2 coat protein and its variants (PP7 system), or to the human U1A protein (7), which directly binds to specific RNA hairpin sequences. However, FPs are less bright and photostable than organic fluorophores (8,9) and require more than 24 copies of MS2 binding sites (accommodating 48 GFPs) to localize single mRNA molecules (10), making the MS2 array a bulky label (200 × 5 × 3 nm) that might perturb mRNA interactions. An alternative approach used live-cell tracking of mRNA molecules singly labeled using organic fluorophores, e.g. by tagging the MS2 coat protein via a polypeptide linker (SNAP tags (11)), or through RNA aptamers (12,13); the latter method has also been used for tracking single mRNA molecules in mammalian cells (14).

The spatial distribution of ribosomal RNA (rRNA) has also been examined, with super-resolution imaging studies in live bacteria showing exclusion of ribosomes from the cell nucleoid (15); in contrast, ribosomal subunits S30 and S50 were shown to diffuse throughout the cell, and not to be excluded from the nucleoid (16).

A third RNA species important for translation is transfer RNA (tRNA), the complement of short and stable RNAs (74–93 nt in length) that translate the nucleotide sequence in an mRNA to the amino-acid sequence of the coded protein. Despite the obvious importance of tRNA in translation, its intracellular mobility and subcellular localization is essentially unknown. A major reason for this gap is the difficulty in labeling tRNA *in vivo*, since incorporation of RNA hairpins for MS2 or aptamer binding may interfere with tRNA interaction with the aminoacyl synthetases, elongation factors or ribosomes. However, tRNA molecules can be labeled *in vitro* by either derivatization of the charged amino acid (17), or via covalent attachment of fluorophores to modified nucleosides with unique chemical reactivity (18–22). The dihydrouridine (DHU) position in the D-loop of tRNA can also be used to attach proflavine (23–26) or to introduce hydrazides for cyanine dye labeling (27). Such labeled tRNA allowed many *in vitro* studies of translation dynamics using single-molecule fluorescence imaging (19,28) and single-molecule FRET (21,29–31); tRNAs labeled with organic fluorophores have also been used to visualize protein synthesis in both fixed and live mammalian cells through FRET signals generated when labeled tRNAs bound at adjacent ribosomal sites (32,33).

Here, we studied the intracellular diffusion and subcellular distribution of tRNA using internalization of fluorescent-labeled tRNA (fl-tRNA) into live *Escherichia coli* through an electroporation-based method recently developed in our lab (34). Using single-particle tracking (35–37), we traced individual tRNA molecules in live cells and observed two modes of diffusion: a fast mode consistent with free tRNA, and a slow mode consistent with tRNAs bound to ribosomes. tRNA molecules displayed a peripheral distribution in cells, wherein fast diffusing tRNA molecules were enriched at the nucleoid, and slow diffusing tRNA molecules localized predominantly at the cell periphery (similar to ribosomes). Antibiotic treatments and controls demonstrated that the uneven tRNA distribution patterns were translation-dependent. Our electroporation method is general and paves the way for diverse single-molecule fluorescence studies of a wide variety of RNA molecules in live bacteria.

## MATERIALS AND METHODS

### RNA labeling and purification

Total *E. coli* tRNA (Roche Diagnostics) were labeled at DHU positions with Cy3, Cy5 or Cy5.5 as described in (22,38). In all cases, the tRNA required HPLC purification as described in (32), in order to ensure removal of free dye. After purification, labeled tRNA typically contained 0.82 Cy3/tRNA, 0.80 Cy5/tRNA and 0.67 Cy5.5/tRNA. tRNAs were stored at −80°C in 10 and 1 μM stocks for use in single-cell and single-molecule studies, respectively. Azide-modified 25-nt RNA (azide-AAUUGUGAGAGCGGAUAACAAUUUC), were labeled with DBCO-Sulfo-Cy5 (gift by Alexandra Tomescu and Tom Brown lab) and stored in 1 μM stocks at −20°C for use in single-cell and single-molecules studies.

### Electroporation and sample preparation

Commercially available electrocompetent *E. coli* cells, ElectroMAX DH5α-E Competent Cells (Invitrogen), were used for electroporation. Cells were diluted 1:1 with sterile milli-Q water and stored at −80°C. For each electroporation experiment, 20 μl of electrocompetent cells were used.

Up to 2 μl of labeled tRNA stored in water milli-Q were added to 20 μl electrocompetent cells (to final concentration of 50 nM, 200 nM or 1 μM tRNA in electroporation cuvette). We also added 1 mM of ethylenediaminetetraacetic acid (EDTA) to the mixture of electrocompetent cells and labeled tRNA, which has to proven to stabilize nucleic acids during electroporation (39). The cell suspension was transferred into a prechilled electroporation cuvette (0.1 cm gap cuvette, Bio-Rad) and placed into an electroporator (MicroPulser, Bio-Rad). An electric field of 1.4 kV/cm was applied for electroporation and 500 μl of super optimal broth with catabolite repression (SOC) was added immediately after electroporation. Cells were recovered for 30 min at 37°C. After recovery, cells were harvested by centrifugation at 3300 *g* for 1 min at 4°C and washed five-times with 500 μl phosphate buffered saline (PBS). Cells were resuspended in 80–120 μl PBS and placed on 1% agarose pads before imaging. The agarose pads were made from about 500 μl of M9 medium containing 1% (v:w) BioRad Certified Molecular Biology Agarose on a coverslip. About 8 μl of cells were pipetted onto the pad, and a burned coverslip was added on top. The slide/agar/slide sandwich was inverted and placed on the microscope stage with the side containing the cells closest to the objective. For antibiotic-control studies, 200 μg/ml chloramphenicol (Cam) or 200 μg/ml rifampicin (Rif) was added to SOC medium and cells were recovered for 30 min. Then, cells were washed as described above except for the final dilution, where cells were diluted in 100 μl PBS containing 200 μg/ml Cam or Rif and then transferred to the agarose pad. Cells were fixed by incubating electroporated cells with 3% (v:v) paraformaldehyde for 1 h after recovery.

### Live-cell imaging

Single-cell fluorescence microscopy in live bacteria was performed on a customized inverted Olympus IX-71 microscope equipped with two lasers, a 637-nm diode laser (Vortran Stradus, Vortran Laser Technology) and a 532-nm DPSS laser (MGL-III-532-100mW, CNI). Laser light was combined into a single-mode optical fiber (Thorlabs) and collimated before focusing on the objective.

Cells were imaged using HILO illumination (40) by adjusting the position of the focused excitation light on the back focal plane of the objective. Typical excitation powers were 20–30 W/cm^2^ for single-cell fluorescence studies (internalization and counting studies) at 50 ms exposure time using 532 and 637-nm laser lines. For single-molecule tracking studies, laser powers at 637 nm of 400 W/cm^2^ were used. Stroboscopic illumination was employed for single-particle tracking, exciting the sample for 1 ms while exposing the camera chip for 5 ms to overcome motion blurring of fast diffusing tRNA molecules. We note that for single-molecule tracking studies, we sometimes pre-bleached entire fields-of-view for up to 5 s before starting data acquisition to diminish the pool of fluorescent tRNA molecules such that we were able to work on the single-molecule level and perform single-molecule localization and tracking analysis (see Supplementary Data).

Cellular and single-molecule fluorescence was collected through the same objective, filtered to remove excitation light through a long-pass filter (HQ545LP, Chroma) and a notch filter (NF02-633S, Semrock), and spectrally separated by a dichroic mirror (630DRLP, Omega). Each channel was imaged onto separate halves of an electron-multiplying charge-coupled device camera chip (iXon+, BI-887, Andor). The illumination for bright-field images comprised a white-light lamp (IX2-ILL100, Olympus) and condenser (IX2-LWUCD, Olympus) attached to the microscope. Movies and images were recorded using the Andor camera software.

### Evaluation of cellular loading

Cells were categorized as loaded if their overall cellular fluorescence per cell area was larger than the mean plus three standard deviation of the overall cellular fluorescence per cell area of non-electroporated cells. Using this internalization threshold, <2% of the cells of non-electroporated and non-treated control samples were classified as loaded. More than 500 cells per sample were imaged and ∼85% of cells were loaded when internalizing tRNA-Cy3, ∼98% of cells with tRNA-Cy5, >60% of cells with tRNA-Cy5.5 and >85% of cells with ssRNA-Cy5. The lower loading efficiency of tRNA-Cy5.5 could be due to inefficient excitation of Cy5.5 on our setup (Cy5.5 at 637 nm: 40% of maximum absorption level, whereas Cy5 at 637 nm: 80% of maximum absorption level) and hence less fluorescence will be detected above the cellular autofluorescence. The excitation of the Cy5.5 cyanine dye could be further improved, but we wanted to keep laser powers at 637-nm the same for Cy5 and Cy5.5 excitation for better comparison of their photobleaching lifetimes (Supplementary Figure S4).

### Single-cell photobleaching studies and counting of tRNA molecules per cell

We measured the fluorescence decay of entire field-of-views containing 30–50 cells under continuous wave illumination in the respective fluorescence channel. Cells were segmented automatically by adapting the MATLAB implementation ‘Schnitzcells’ (41) for brightfield cell images. The manually adjusted cell masks were used to extract cells’ fluorescence data by calculating the total fluorescence intensity per cell area within each cell mask for each movie frame. Cell autofluorescence per cell area after photobleaching was subtracted using a custom-written MATLAB script (Mathworks). This baseline subtraction was routinely performed for all single-cell fluorescence time-traces but we note that for photobleaching studies a single exponential fit (+offset) to the single-molecule fluorescence time-trace is sufficient to obtain the photobleaching life-time. Here, baseline-subtracted photobleaching time-traces of cells from single-cell studies (1 μM tRNA-Cy5 in electroporation cuvette, Figure 2D) were fitted with a single exponential to obtain the cell-based photobleaching lifetime as a measure of fluorophore photostability.

Baseline-subtracted photobleaching time-traces of cells from single-molecule studies (∼50 nM of tRNA-Cy5 in electroporation cuvette, Figure 2D), which showed <6 quantized steps, were fitted with a hidden Markov model (HMM) as described in Ref. (34) to the unitary intensity. Step heights recovered by the HMM algorithm from 158 photobleaching time traces per sample were plotted in the step height histogram. The step-height histogram was fitted with a single one-dimensional-Gaussian (free fit parameters: position1, width1, amplitude1). The center of the fitted Gaussian peak corresponds to the fluorophore unitary intensity, a measure of the fluorophore brightness. The unitary intensity of (8.7 ± 2.4) a.u. was obtained for Cy5, which corresponds to (7500 ± 2100) photons/s. The number of internalized tRNA molecules per cell was calculated by dividing the overall cellular fluorescence to the fluorophore unitary intensity.

### Single-molecule localization and single-particle tracking

Custom MATLAB (Mathworks) software was used to analyze single-molecule tracking and diffusion in live *E. coli* as described in (34) and (37). Briefly, the PSFs in each movie frame were fitted by a 2D elliptical Gaussian (free fit parameters: x/y position, x/y width, elliptical rotation angle, amplitude, background) using initial position guesses from applying a fixed localization-intensity threshold on the bandpass filtered fluorescence image (42)—software available from our lab website (https://groups.physics.ox.ac.uk/genemachines/group/Main.Software.html). Single-molecule tracking was performed by adapting the MATLAB script based on a published algorithm (43). Localized PSFs were linked to a trajectory if they appeared in consecutive frames within a window of 7 pixels (∼0.67 μm). This window size ensures 98% of steps are correctly linked for a single diffusive species with an apparent diffusion coefficient (*D*_app_) of 4 μm^2^/s and 5 ms exposure time. To account for transient disappearance of the PSF within a trajectory due to blinking or missed localization, we used a memory parameter of one frame.

A detailed description of the single-particle tracking analysis and the calculation of the cumulative distribution function (CDF), apparent *D*_app_ and fitting routines of the CDF curves and *D*_app_-histograms is presented in detail in the Supplementary Data. Since the apparent *D*_app_ does not take cellular confinement, motion blurring and localization imprecision (37,44) into account, we simulated raw movies of diffusing fluorescent molecules in a rod-shaped cylinder to relate the apparent *D*_app_ to an accurate *D*_app_; further details are presented in the Supplementary Data.

### tRNA spatial distribution analysis

Analysis was performed in MATLAB. Cells were segmented from bright-field images using MicrobeTracker (45), obtaining a cell outline and cell midlines. The positions of the molecule trajectories were determined relative to the cell midline, with the x-axis defined as the cell long axis and the y-axis as the cell short axis. The distances were normalized to 0 (cell midline) and 1 (cell outline). For the probability distributions plots, localizations were normalized by their relative position along the short cell axis and along the long cell axis (only absolute distance).

The analytical probability distribution for a uniform distribution within a cylindrical volume is given by:

\begin{equation*}pdf\left( \rho \right) = \frac{{4\ \sqrt {{\rm{max}}\left( {{r^2} - {\rho ^2},\ 0} \right)} }}{\pi }\end{equation*}

where ρ is the radial coordinate and r is the cylinder radius, for the unit cell holds *r* = 1.

Two-dimensional histograms of the spatial distribution of tRNA molecules throughout the cell cycle were obtained by binning cells according to cell length (short cells: 1.7–2.9 μm and long cells: 3.1–4.3 μm) with short cells having a single nucleoid located in the centre of the x-axis and and long cells having two nucleoids (37).

## RESULTS

### Efficient internalization of organic dye-labeled RNAs into live *E. coli* cells

We first tested whether short RNAs labeled with organic fluorophores could be internalized efficiently into live *E. coli* using electroporation, as in previous studies showing efficient internalization of 45-bp ssDNA and dsDNA, as well as proteins of up to 100 kDa (34,46–48). We reasoned that RNAs shorter than ∼100 nt should be small enough to enter the cell during electroporation (34); such RNAs also carry enough net negative charge to avoid any sticking to the cell exterior (47). Additionally, we stabilized the RNA by adding 1 mM EDTA to the electroporation buffer, since this addition has proven to stabilize and internalize DNA into live bacteria (39). In terms of fluorophores, we initially selected Cy3, Cy5 and Cy5.5, due to their high photostability and brightness (46).

**Figure 1.**
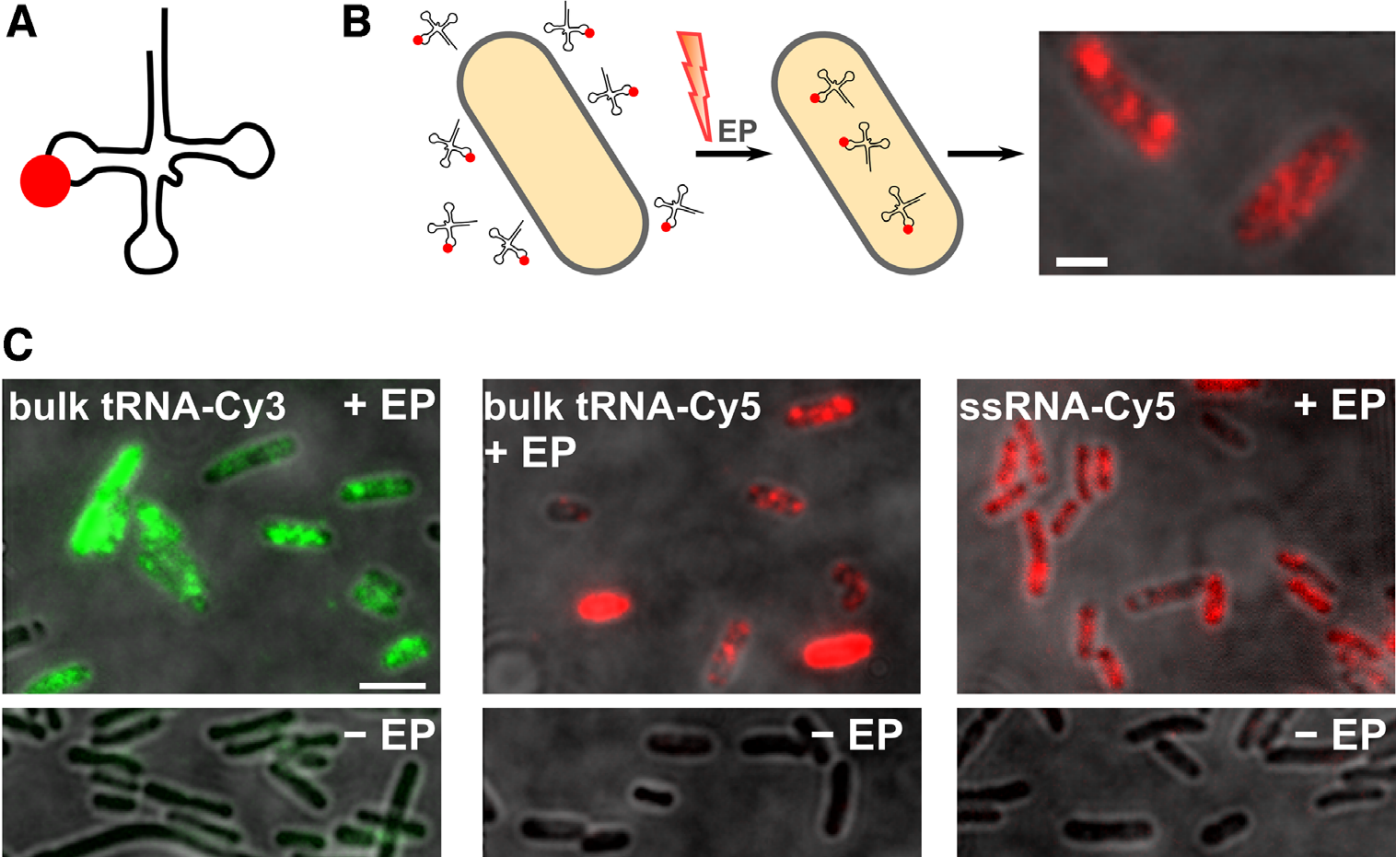
Internalization of labeled tRNA molecules into live *Escherichia coli*. (**A**) Schematic of labeled tRNA molecule. (**B**) Schematic of the internalization method using electroporation (EP). Labeled tRNA molecules are added to cell suspension of electrocompetent cells and are exposed to the discharge of a high-voltage electric field. Cells are quickly recovered and non-internalized tRNA molecules are washed off. Cell imaging is performed on an inverted microscope using HiLO illumination. Scale bar: 1 μm. (**C**) Efficient loading of *E. coli* cells with tRNAs labeled with organic fluorophores of different spectral range. Cells show high inner-cell fluorescence upon electroporation with 1 μM of tRNA-Cy3, tRNA-Cy5, and ssRNA-Cy5 (top row, left to right) and basal fluorescence of non-electroporated cells corresponding to cellular autofluorescence in the respective spectral channel (‘−EP’ cells, bottom row). More than 85% of cells were loaded when internalizing tRNA-Cy3, tRNA-Cy5 and ssRNA-Cy5 (>500 cells per dataset). Scale bar: 3 μm.

The RNA molecules delivered were uncharged bulk *E. coli* tRNA (hydrodynamic radius, *R*_h_ = 2.6 nm, Supplementary Data), and a 25-mer ssRNA (‘RNA25’). Organic dye-labeled bulk *E. coli* tRNA molecules (Figure 1A), as well as RNA25 were electroporated into electrocompetent *E. coli* DH5α cells (‘Materials and Methods’ section) and imaged under highly-inclined and laminated optical sheet illumination (40) in the respective fluorescent channel (Figure 1B). Electroporation conditions were optimized (‘Materials and Methods’ section) to maximize both RNA integrity and cell viability, with ∼70% of cells growing and dividing after electroporation (46) (Supplementary Figure S1).

In all cases (bulk tRNA labeled with Cy3, Cy5, or Cy5.5 and RNA25-Cy5), RNA was efficiently internalized into bacteria using electroporation (Figure 1C). For all samples, electroporated cells showed higher intra-cellular fluorescence than non-electroporated cells or electroporated cells without labeled RNA added (Figure 1C and Supplementary Figure S2). The internalization efficiency was calculated relative to non-electroporated cell control (to which labeled RNAs were added, *not* electroporated, washed and recovered like the electroporated sample), with >85% of cells showing uptake of tRNA-Cy3, tRNA-Cy5 and RNA25-Cy5 (Figure 1C, left to right) and >60% of cells showing uptake of tRNA-Cy5.5 (Supplementary Figure S2).

### Delivery of >100 labeled tRNA molecules into bacterial cells

**Figure 2.**
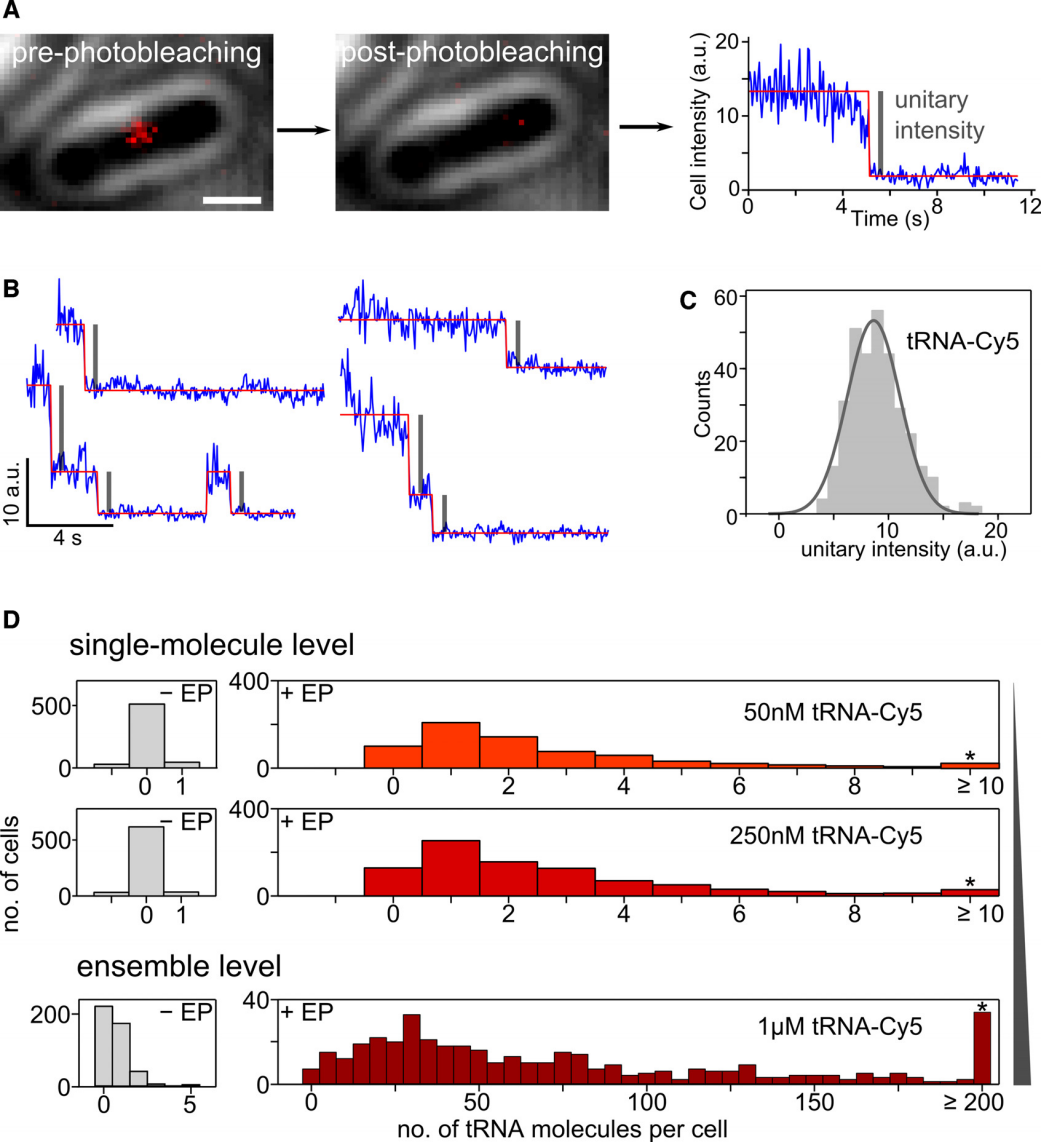
Counting and controlling the number of internalized tRNA molecules per cell. (**A**) Single-cell photobleaching step analysis of tRNA-Cy5 in live bacteria. Scale bar: 1 μm. (**B**) Photobleaching time-trace samples (blue) and Hidden–Markov Model fit (red) of single photobleaching steps corresponding to the single-molecule unitary intensity (gray bars). (**C**) Step height histogram (unitary intensity distribution) of tRNA-Cy5 (158 photobleaching time-traces, 324 fitted single photobleaching steps = unitary intensity) and Gaussian fit centered at (8.7 ± 2.4) a.u. corresponding to Cy5 brightness of (7500 ± 2100) photons/s. (**D**) Counting the number of internalized tRNA-Cy5 molecules per cell at different concentrations of labeled tRNA-Cy5 in cell suspension before electroporation. Cells are loaded with 1–10 tRNA molecules when adding 50 nM tRNA-Cy5 (mean: 1.4 molecules per cell, median: 0.9) and up to 250 nM tRNA-Cy5 (mean: 1.5 molecules per cell, median: 1.0) in the cell suspension before electroporation, which is an ideal regime for single-molecule studies. Cells are loaded with about 100 tRNA molecules per cell (mean: ∼140, median: ∼100, Supplementary Figure S5 for tRNA-Cy3 and tRNA-Cy5.5) at 1 μM tRNA-Cy5 initial concentration before electroporation.

We counted the number of internalized tRNA molecules per cell using single-cell photobleaching (46). To obtain the unitary intensity corresponding to each internalized fluorescent tRNA, we first photobleached cells to the point where only few (*N* < 10) fluorescent RNAs per cell remained, measured the decay of the overall cellular fluorescence in individual cells and obtained single-cell photobleaching time-traces (Figure 2A). The photobleaching time-trace shows distinct single-step decreases of the overall cellular fluorescence, attributable to the photobleaching of a single fluorophore (blue single-cell intensity time-trace, Figure 2A) and are used to relate the number of photobleaching steps to the number of internalized tRNA-Cy5 molecules per cell. We then fitted the photobleaching time-traces using HMM (‘Materials and Methods’ section) and obtained the unitary intensity for each photobleaching step (HMM fit in red, gray bar highlights unitary intensity, Figure 2A). Single-cell photobleaching time-traces from tRNA-Cy5 experiments (50 nM tRNA-Cy5 in electroporation buffer) showed distinct step-like behavior (*N* = 158, Figure 2B), and HMM analysis revealed the internalization of 1–3 molecules per cell (Figure 2B and Supplementary Figure S3a). The unitary intensity for tRNA-Cy5 was 8.7 ± 2.4 a.u., corresponding to Cy5 brightness of ∼8000 photons/s (Figure 2C); the tRNA-Cy3 and tRNA-Cy5.5 were less bright (Supplementary Figure S3b). The photon budget of a fluorophore is important for accurate localization of a tRNA molecule, since the localization precision is inversely proportional to the square-root of the total photon count (49).

Next, we used single-cell photobleaching of cells loaded with >100 molecules to measure the decay of fluorescence over time and obtained photobleaching lifetimes for the different fluorophores (Supplementary Figure S4, Cy5: 3.4 ± 1.1 s, Cy3: 21.4 ± 5.8 s and Cy5.5: 4.6 ± 1.5 s). Since the lifetimes were in the range of several seconds, and since a tRNA spends only a fraction of a second in a ribosome during translation (prokaryotic translation rates are 10–20 aa/s (50,51)), the available observation time window should allow the observation of tRNAs on a ribosome during polypeptide synthesis, something difficult for FP due to their limited photostability (52).

We next used the measured unitary intensity to relate the overall cellular fluorescence to the number of internalized labeled tRNA molecules per cell. To determine the molecular density in cells that will allow us to localize single tRNA molecules, we evaluated different initial concentrations of labeled tRNA-Cy5 before electroporation (i.e. in the buffer surrounding the cells in the electroporation cuvette) by counting the number of tRNA molecules that were eventually internalized per cell. We observed a wide distribution of internalized molecules per cell (Figure 2D), and identified two working regimes: a ‘single-molecule regime’, with 1–3 internalized labeled molecules/cell (50–250 nM tRNA-Cy5: mean ∼1.5, median ∼1.0) and an ‘ensemble regime’, with up to hundreds of internalized molecules per cell (at 1 μM tRNA-Cy5: mean ∼140, median ∼100; see also Supplementary Figure S5 for tRNA-Cy3 and tRNA-Cy5.5).

### The diffusion profile of tRNA in live *E. coli*

**Figure 3.**
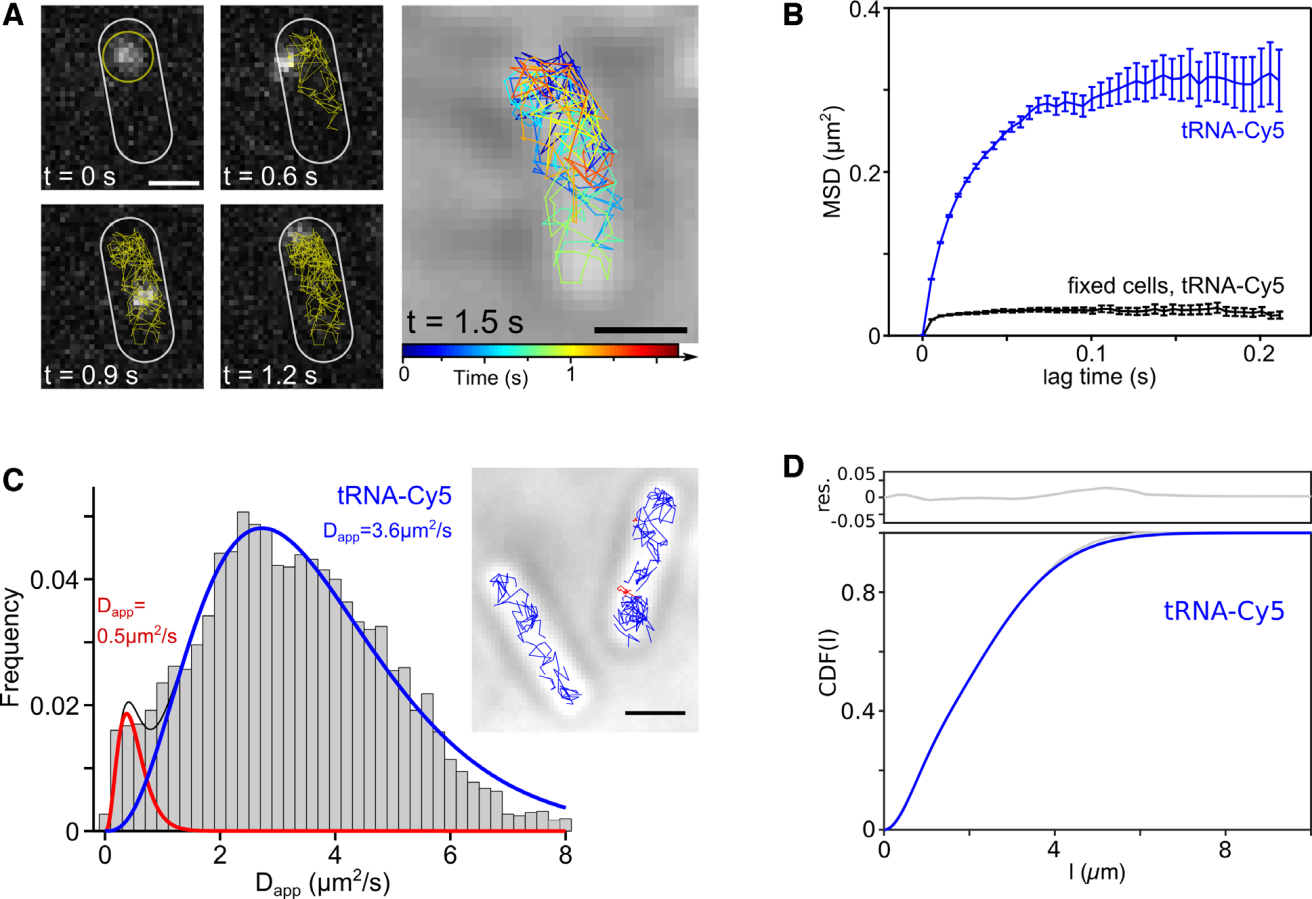
Single-molecule tracking of tRNA-Cy5 and measurement of diffusion coefficient (*D*_app_) of tRNA *in vivo*. (**A**) Tracking of a single tRNA-Cy5 molecule for ∼1.5 s and reconstruction of the single-molecule trajectory in the bacterial cell (90 FOV, 700 cells and ∼20 000 localizations for live-cell dataset and ∼200 localizations for negative controls −tRNA + EP and +tRNA − EP). Scale bar: 1 μm. (**B**) Measurement of the mean-squared displacement (MSD) for tRNA-Cy5 in live cells (blue) and fixed cells (black). The MSD plateaus due to cellular confinement and motion blurring in live cells and due to the localization precision. We calculated the localization precision σ from the apparent *D*_app_ of the fixed cell control *D*_app_(fixed cells) = 0.32 μm^2^/s = σ^2^/(4Δt), with Δt = 5ms and obtained σ = 0.08 μm, Supplementary Figure S6b. (**C**) Apparent *D*_app_ distribution of tRNA-Cy5 and fit to two diffusive species. The slow diffusive species was constrained to ribosomal complex diffusion of *D*_app_(ribosome) = 0.5 μm^2^/s (red, 5%, Ref (26)) and free fit of fast diffusing species resulted in *D*_app_(tRNA-Cy5) = 3.6 μm^2^/s (blue, 95%). Similar fitting results were obtained when constraining the slow diffusing species to immobile molecules of the fixed-cell control, *D*_app_(fixed cells) = 0.32 μm^2^/s (Supplementary Figure S6b, c and g) or when allowing a free fit to one, two and three diffusive species (Supplementary Figure S6d–f). Including another independent dataset (90 FOV, 582 cells), the apparent *D*_app_ of tRNA in live cells was obtained to *D*_app_(tRNA-Cy5) = (3.5 ± 0.2) μm^2^/s (Supplementary Figure S6h). (**D**) Cumulative distribution function (CDF) of tRNA-Cy5 tracking data was freely fitted to two diffusive species and the *D*_app_ of the fast diffusing species was obtained to D_2_ = 7.6 μm^2^/s (slow diffusive species: D_1_ = 0.12 μm^2^/s).

We chose the single-molecule regime to study the localization and diffusive behavior of tRNA in the bacterial cytoplasm. We expected the diffusion of unbound tRNA-Cy5 molecules to be in the 6–13 μm^2^/s range, due to the similarity in the hydrodynamic radii of tRNAs and GFP (tRNA: 2.6–2.8 nm (53,54), GFP: 2.4 nm (55); Supplementary Data). To capture fast diffusion modes, we used stroboscopic illumination (using 1-ms laser excitation pulses during 5-ms camera exposures) to obtain bright diffraction-limited images of single tRNA molecules within live bacterial cells (Figure 3A, *t* = 0 s). Each single tRNA molecule was localized in each frame, and the localizations within consecutive frames were linked to generate a single-molecule trajectory (Figure 3A, yellow trajectory at *t* = 0.6, 0.9, 1.2 s, ‘Materials and Methods’ section).

The trajectories illustrated that tRNA molecules explore most of the cellular environment over time (Figure 3A, Supplementary Figure S6a and Supplementary Movies 1 and 2). Using a large number of single-molecule trajectories (*N* = 4122), we calculated the mean-squared displacement (MSD) for tRNA molecules tracked in more than three consecutive frames. The MSD of tRNA-Cy5 *in vivo* (Figure 3B, blue) plateau due to cellular confinement and localization precision (44). We estimated the localization precision under these experimental conditions to 80 nm using a fixed-cell control (Figure 3B, black).

We next calculated the distribution of apparent *D*_app_ from the MSDs of individual trajectories truncated to four steps, (37,52) and Supplementary Data. The *D*_app_ histograms for tRNA-Cy5 show a bimodal distribution (Figure 3C and Supplementary Figure S6e, g and h), and do not fit to an analytical distribution of a single diffusive species (Figure 3C). We reasoned that tRNA molecules could be either freely diffusing; or slowly diffusing when being bound to moderate-size proteins (e.g. either to tRNA-synthetase or to the elongation factor EF-Tu as a ternary complex of tRNA–EF-Tu–GTP), or to the ribosomes during translation (19). To explore these options, we fitted the *D*_app_-distribution to two γ-distributions for two diffusive species, with the slow diffusing species set to the *D*_app_ expected for the assembled ribosome (*D*_app_ = 0.5 μm^2^/s; see ‘Discussion’ section) and the fast diffusing species left free; this fit (Figure 3C) recovered a major (∼90%) fast diffusing species with *D*_app_ ∼3.6 μm^2^/s, which indicates considerable mobility for tRNA. We also fitting the *D*_app_-distribution freely fitted to one to three species (corresponding to ribosomal diffusion and/or to ternary-complex diffusion) and still obtained a major (∼70%) mobile population (Supplementary Figure S6d–f). The apparent *D*_app_ from the free fits of the *D*_app_-distribution to two or three species ranged from *D*_app_ = 3.3 ± 1.6 μm^2^/s (free, two species), D_app_ = 3.5 ± 1.7 μm^2^/s (free, three species) and shows that the major tRNA species was fast diffusing similar to the apparent *D*_app_ obtained from constrained fits of the slow diffusing species to ribosomal and ternary complex diffusion *D*_app_ = 3.4 ± 1.7 μm^2^/s (constrained, two species), *D*_app_ = 3.7 ± 1.8 μm^2^/s (constrained, three species), Supplementary Figure S6g–i. We estimated the *D*_app_ of the ternary complex to be ∼2.4 μm^2^/s under our experimental conditions using diffusion simulations (Supplementary Figure S7a), since the accurate *D*_app_ of the protein Fis (with similar size of the ternary complex (56)) was determined to D∼4.5 μm^2^/s (37). Including more data sets for bulk tRNA and testing several fitting modes (single species, constrained to fixed cell control, constrained to ternary complex and ribosomal diffusion, Supplementary Figure S6g–i) we obtained a *D*_app_(tRNA-Cy5) = 3.5 ± 0.2 μm^2^/s for the mobile species. We note that data sets taken on different days show different proportion of slow diffusing molecules (5–11%), which could be due to variability of the electroporation process and cellular uptake. However, even when fitting the *D*_app_ distribution to two or three diffusive species, we always obtained a major fast diffusing species (>70%) that matched the expected value for freely diffusing tRNA molecules (Figure 3C and Supplementary Figure S6d–i).

Since the apparent *D*_app_ is affected by experimental conditions (cellular confinement, motion blurring, localization precision), we simulated single-molecule tracking movies to relate the measured *D*_app_ to accurate *D*_app_ *in vivo*. We simulated free Brownian motion of single tRNA molecules in a confined cellular environment mimicking similar experimental conditions and performed the same single-molecule tracking analysis as for experimental data (Supplementary Data; ∼1.5 molecules per cell, 500 cells for each simulation run, Supplementary Movie 3 and 4). This calibration of the measured *D*_app_ with the actual *D*_app_ input for the simulations (Supplementary Figure S7a and b) resulted in an accurate *D*_app_ of D_tRNA_ = 8.1(−1.0 + 0.5) μm^2^/s (Supplementary Figure S7c and d).

### Non-uniform spatial distribution of tRNA molecules *in vivo*

**Figure 4.**
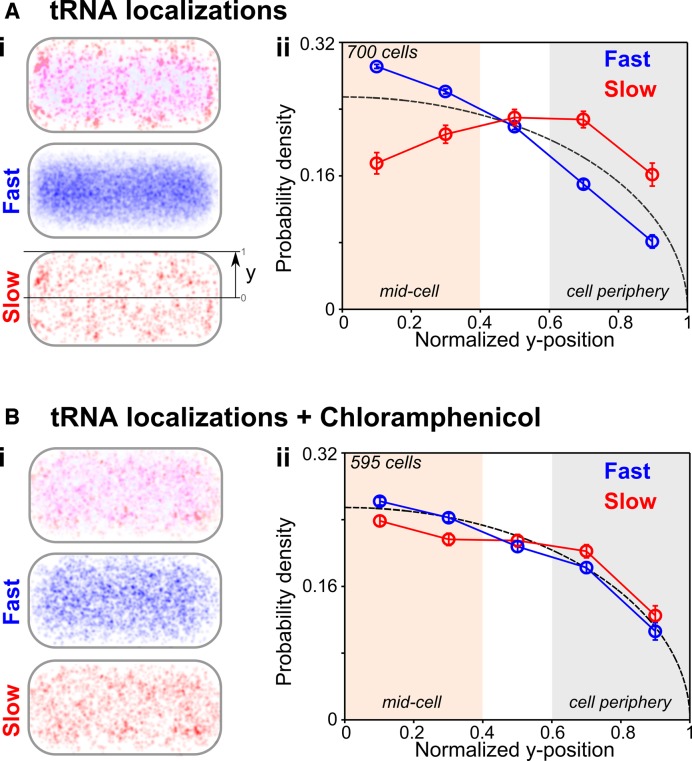
Spatial distribution of tRNA molecules in live bacterial cells. Localizations of tRNA-Cy5 molecules were normalized along cell-length and cell-width and were represented in unit cell (‘Materials and Methods’ section). (Ai) Localizations of tRNA molecules classified as fast diffusing (*D*_app_ > 1 μm^2^/s, blue) and slow diffusing (red) and combined (top) were shown in unit cells, respectively. (Aii) Probability distribution of fast and slow diffusing molecules along cell width (y-axis, short cell axis). Bias of slow diffusing molecules to cell periphery and fast diffusing molecules to mid-cell relative to uniform-distribution (dotted line). (**B**) Spatial distribution of tRNA molecules upon Chloramphenicol treatment blocking translation. (Bi) Representation as in Ai. (Bii) Fast and slow diffusing tRNA molecules were evenly distributed along cell widths. Results in (A and B) indicate that slow diffusing tRNA molecules at the cell periphery were utilized during translation. The spatial distribution of control measurements such as unspecific RNA, and fixed cells are shown along cell within Supplementary Figure S9 and within a unit cell in Supplementary Figure S10 for different cell length.

To determine the tRNA spatial distribution, we plotted the localizations of fast diffusing tRNA molecules (blue) and slow diffusing tRNA molecules (red) within a unit cell, normalizing along cell-length and cell-width (Figure 4Ai). We used a threshold of D < 1 μm^2^/s to select slow diffusing molecules, such that 90% of all trajectories in the fixed-cell control were categorized as slow diffusing (Supplementary Figure S6b and c).

The localization maps show a distinct enrichment of slow diffusing tRNA molecules at the cell periphery (defined as the intracellular space between the nucleoid and the inner membrane), whereas fast diffusing molecules localized with a slight preference for proximity to the mid-cell region, a region occupied by the bacterial nucleoid. This was also evident after plotting the probability density of slow and fast diffusing molecules along the short axis of the unit cell (Figure 4Aii, top: unit cell showing cell periphery (gray) and mid-cell (beige)); slow diffusing tRNA molecules were enriched at the cell periphery, and were partly excluded from the mid-cell region (Figure 4Aii, slow diffusing species (red) relative to uniform distribution (dotted line): −24% at mid-cell and +33% at cell periphery). Furthermore, the fast diffusing localized predominantly along mid-cell (Figure 4Aii, fast diffusing species (blue) relative to uniform distribution (dotted line): +10% at mid-cell and −19% at cell periphery), as previously observed for the nucleoid-associated protein HU (16,52).

To dissect further the exclusion of slow diffusing tRNA molecules from the mid-cell region and the nucleoid, we sorted cells along cell length (cell length distributions in Supplementary Figure S8). Specifically, we looked at short cells (1.7–2.9 μm long), which are expected to have only a single chromosome) and long cells (3.1–4.3 μm long), which contain a chromosome that has been largely duplicated (dataset 1 and Supplementary Figure S10). For short cells, we again observed a clear tendency of slow diffusing molecules to localize at the cell periphery (especially close to the cell endcaps), while fast diffusing molecules explore predominantly the mid-cell region (Supplementary Figure S10).

The spatial distribution of fast diffusing molecules within long cells was similar to that of short cells, apart from the appearance of two local maxima along the long cell-midline; this latter feature suggests the presence of two chromosomes, from which slow diffusing tRNA molecules were largely absent, again enriching the cell periphery (Supplementary Figure S10). This further indicates that mobile tRNA molecules could diffuse freely and explore the entire nucleoid, whereas slow diffusing tRNA molecules localize at the cell periphery and are not binding significantly within the nucleoid interior. Notably, the 12% fraction of slow-to-fast diffusing tRNA molecules (using a simple *D*_app_ threshold) does not change throughout the cell cycle (since we observe 13 and 11% for this fraction in short and long cells, respectively) and is in good agreement with the result from our *D*_app_-distribution fit (Figure 3C).

### Blocking translation changes the tRNA localization for slow diffusing molecules

The peripheral localization pattern of slow diffusing tRNA throughout the cell cycle is consistent with the internalized tRNA being utilized in protein synthesis, since previous studies showed the exclusion of ribosomal complexes from the nucleoid (16). To test this hypothesis, we treated cells with the antibiotic chloramphenicol (Cam, which binds to the 50S ribosomal subunit, preventing tRNA accommodation at the A-site and inhibiting peptide bond formation (57)), and repeated the analysis (Figure 4Bi). Indeed, chloramphenicol treatment resulted in a much more uniform distribution for the slow diffusing tRNA, with loss of the cell-periphery bias (with the fraction of molecule in the periphery decreasing from +33 to +14%) (Figure 4Bii); this loss was seen for all cell lengths (Supplementary Figure S10). The new picture after chloramphenicol treatment shows that both slow and fast diffusing tRNA molecules localize evenly throughout the entire cell. These results suggest that the peripheral tRNA localization pattern for slow diffusing tRNA was due to their binding to ribosomes with peripheral localization (see ‘Discussion’ section).

Further evidence of translation-dependent localization of slow diffusing tRNA molecules at the cell periphery was obtained by studying the spatial distribution of tRNA-Cy5 molecules in fixed cells; non-specific ssRNA-Cy5 (RNA25) molecules *in vivo*; and through Monte-Carlo diffusion simulations (Supplementary Figure S9). In all three controls, the internalized molecules were uniformly distributed within the cell and no peripheral localization pattern was observed.

## DISCUSSION

### Detection and localization of small RNAs in live bacteria

Using an electroporation-based technique, we efficiently internalized fluorescent RNA molecules into live bacteria. The internalization of RNA labeled with bright and photostable organic-dyes opens new ways for studying many forms of stable RNAs (tRNAs, rRNA fragments) and regulatory RNAs (riboswitches and 5S RNA). Labeling with small organic fluorophores is expected to be much less perturbing than the introduction of ∼1000-fold larger probes based on FP. The use of fluorophores such as Cy3, Cy5 or Cy5.5 opens a wide spectral range (500 to 700 nm) for *in vivo* fluorescence studies. This range has the benefit of lower cellular autofluorescence, especially in the red and infrared spectrum (58,59). Apart from the novel single-molecule sensitivity (tracking of individual tRNAs) described here, the internalization of molecules at the small-ensemble regime (10–100 molecules per cell) should enable other fluorescence imaging techniques, such as fluorescent recovery after photobleaching (FRAP) and fluorescence correlation spectroscopy (FCS) studies.

### Single-particle tracking shows that tRNA exhibits mainly free diffusion in *E. coli*

Despite its obvious importance, the diffusion properties of tRNA *in vivo* were poorly characterized in all organisms, including bacteria. Early measurements in cell lysates yielded an estimate of D of ∼60 μm^2^/s *in vitro* (60), whereas diffusion simulations using a bead-model of tRNA estimated a much lower value (D∼10 μm^2^/s; (61)). Due to the similar size of tRNA molecules and GFP (hydrodynamic radius of 2.6 and 2.4 nm for yeast tRNA^Phe^ (53,54) and GFP (55), respectively), we expected that, if tRNA diffuses freely *in vivo*, its *D*_app_ will be only ∼10% smaller than for GFP. The GFP *D*_app_ was in the range of 6–10 μm^2^/s using FCS and FRAP (62); however, single-molecule tracking studies of FP such as mCherry, Venus, Dendra2 and mEos2 obtained slightly higher *D*_app_ (7–13 μm^2^/s) in the *E. coli* cytoplasm ((16,36,52,63), Supplementary Data). Hence, the *D*_app_ of 8.1 ± 1.0 μm^2^/s for tRNA (Figure 3C and D, Supplementary Figure S6d–h and simulations Supplementary Figure S7) is in excellent agreement with the values expected from free diffusion.

Based on the high equilibrium association constant (7 × 10^6^ M^−1^; (64)) of aa-tRNA binding to EF-Tu-GTP (ternary complex), and the observation in eukaryotic cells of ‘tRNA channeling’ (a process where tRNA is directly transferred from tRNA synthetases to elongation factors and then to ribosomes without dissociating; (65)), we tested the prediction of such a mechanism in *E. coli* by fitting the apparent *D*_app_ distribution with two constrained species (ribosomal diffusion and ternary-complex diffusion). However, we still obtained about 70% of a fast diffusing species (with *D*_app_ > 3.5 μm^2^/s).

Our results show that a large fraction of internalized fluorescent tRNA (>70%) appears to diffuse freely in the bacterial cell. This result is surprising, since it has often been assumed (but has never been shown) that most tRNA is found in a bound state in the cell, either as a complex with tRNA synthetase, or a ternary complex with elongation factor EF-Tu and GTP, or engaged in the ribosome during translation.

We considered possible trivial reasons for the high mobility of internalized fluorescent tRNA. One possibility is that the labeled tRNA may have reduced functionality; however, *in vitro* studies showed that the labeled tRNA is charged normally with amino acids, and that charged-and-labeled tRNA efficiently binds to elongation factor EF-Tu (22). A second possibility is that the binding of electroporated tRNA to tRNA-synthetase is prevented by preferential binding of endogenous tRNA by tRNA-synthetases (as part of a bacterial tRNA channeling process), as shown for electroporated tRNAs in eukaryotic cells (65). However, there is no evidence for any tRNA channeling in bacteria; in fact, bacterial tRNA-synthetases (66) lack tRNA-binding domains, which are present in most eukaryotic tRNA-synthetases and dictate their much more stable tRNA binding. A third possibility is that the electroporation process has changed cell physiology to a degree that it led to tRNA dissociation from its protein or ribosomal partners; since 70% of the cells grow and divide normally after electroporation, deposition and imaging, we consider that this possibility is unlikely to be the main factor for the large fraction of freely diffusing tRNA we observe.

Another consideration is that many of our expectations regarding interaction equilibria *in vivo* come from either *in vitro* studies or indirect cellular studies. For example, although EF-Tu may be abundant in *E. coli*, its potential interactions with actin-like protein MreB (shown in *Bacillus subtilis* and *E. coli* (67,68)) will decrease significantly the amount of free EF-Tu in cells. Further, the *in vivo* affinity of tRNA for its cellular interaction partners (tRNA synthetase, EF-Tu-GTP) is currently unknown and may be weaker than measured *in vitro*.

Given these considerations, our results strongly suggest that most tRNA molecules diffuse freely in cells, supporting a simple mechanism for locating tRNA interaction partners within bacteria; such as a ‘diffusion-based tRNA’ cycle in bacteria (due to the lack of subcellular structure and higher tRNA concentration in prokaryotes compared to eukaryotes) has already been proposed (66).

We also examined whether a diffusion-based tRNA cycle (where tRNAs spend most of their time searching for ribosome using free diffusion and participating in peptidyl transfer reaction in the ribosome) is fast enough to sustain the speed of protein synthesis. Assuming translation rates of 50 ms per amino acid (50) with two tRNA molecules (endogenous tRNA pool: 375 000) present at all 50 000 ribosomes per *E. coli* cell (69,70), we expect 100 000 tRNA molecules bound to ribosomes and 275 000 molecules searching for ribosome sites, resulting to a ribosome-bound tRNA fraction of ∼25%. The fraction also reflects the fraction of tRNA cycle time dedicated to ribosome binding; since the ribosome binding time for a tRNA is 2 × 50 ms = 100 ms, the total cycle is ∼400 ms and the search-and-charging time is ∼300 ms. Indeed, a tRNA molecule can explore the entire cell within 70 ms assuming three-dimensional free diffusion with D_tRNA_ = 8 μm^2^/s and *E. coli* cell dimensions of 3 × 1 μm: MSD(t) = 6D_tRNA_ *t* ≥ 3 μm^2^ → *t* ≥ 70 ms, which is ∼25% of the time the tRNA is allowed to recharge and search for the ribosome assuming a diffusion-based tRNA cycle and rapid cell growth. This calculation shows that the tRNA diffusion is not rate-limiting for protein synthesis in bacteria.

### Peripheral distribution of slow diffusing tRNA molecules

We observed substantial enrichment of slow-diffusing tRNA at the periphery compared to uniform distribution; this enrichment was more apparent when cells were sorted according to size. We propose that tRNA molecules classified as ‘slow diffusing’ associate with ribosomes, since the slow diffusing species matched an apparent *D*_app_ distribution of 0.3–0.5 μm^2^/s (Supplementary Figure S7), consistent with ribosomal diffusion (36); further, slow diffusing tRNA molecules were biased toward the cell periphery, consistent with the observed exclusion of ribosomes from the cell nucleoid (15,71). Alternative interpretations of the data include tRNA complexation with other large macromolecular assemblies.

Our results also show that the fast diffusing tRNA molecules diffuse freely within the entire nucleoid. Along with studies showing that ribosomal subunits S30 and S50 can penetrate the entire cell and are not excluded from the nucleoid (16), our tRNA diffusion and localization results provide further support that heavily transcribed and translated genes move to the nucleoid periphery, as suggested for co-transcriptional translation.

The ∼10% slow-diffusing fraction of tRNA is within the expected values for bacteria with a division time of ∼30 min. Considering that ∼2 internalized labeled tRNA-Cy5 molecules per cell compete with the intrinsic pool of ∼375 000 unlabeled tRNA for maximum two binding sites at ∼50 000 ribosomes per cell (69,70), we estimate an upper limit of 25% of slow diffusing tRNA molecules at the ribosome (based on slow diffusing tRNA = number of ribosomal sites/total amount of tRNA).

Following blocking translation with chloramphenicol (which also has indirect effects due a decrease in the rate of ribosome biogenesis (72)), the periphery bias of slow diffusing tRNAs is drastically reduced (+14% at the cell periphery with Cam versus +33% without Cam), supporting our proposal that tRNA molecules are slowly diffusing with ribosomal complexes outside the nucleoid (15,16).

## References

[1] Buxbaum A.R., Haimovich G., Singer R.H. (2015). In the right place at the right time: visualizing and understanding mRNA localization. Nat. Rev. Mol. Cell Biol..

[2] Eliscovich C., Buxbaum A.R., Katz Z.B., Singer R.H. (2013). mRNA on the move: the road to its biological destiny. J. Biol. Chem..

[3] Long R.M., Singer R.H., Meng X., Gonzalez I., Nasmyth K., Jansen R.P. (1997). Mating type switching in yeast controlled by asymmetric localization of ASH1 mRNA. Science.

[4] Nevo-Dinur K., Nussbaum-Shochat A., Ben-Yehuda S., Amster-Choder O. (2011). Translation-independent localization of mRNA in E. coli. Science.

[5] Femino A.M., Fay F.S., Fogarty K., Singer R.H. (1998). Visualization of single RNA transcripts in situ. Science.

[6] Pitchiaya S., Heinicke L.A., Custer T.C., Walter N.G. (2014). Single molecule fluorescence approaches shed light on intracellular RNAs. Chem. Rev..

[7] Brodsky A.S., Silver P.A. (2002). Identifying proteins that affect mRNA localization in living cells. Methods.

[8] Shaner N.C., Steinbach P.A., Tsien R.Y. (2005). A guide to choosing fluorescent proteins. Nat. Methods.

[9] Dempsey G.T., Vaughan J.C., Chen K.H., Bates M., Zhuang X. (2011). Evaluation of fluorophores for optimal performance in localization-based super-resolution imaging. Nat. Methods.

[10] Fusco D., Accornero N., Lavoie B., Shenoy S.M., Blanchard J.M., Singer R.H., Bertrand E. (2003). Single mRNA molecules demonstrate probabilistic movement in living mammalian cells. Curr. Biol..

[11] Carrocci T.J., Hoskins A.A. (2014). Imaging of RNAs in live cells with spectrally diverse small molecule fluorophores. Analyst.

[12] Dolgosheina E.V., Jeng S.C., Panchapakesan S.S., Cojocaru R., Chen P.S., Wilson P.D., Hawkins N., Wiggins P.A., Unrau P.J. (2014). RNA mango aptamer-fluorophore: a bright, high-affinity complex for RNA labeling and tracking. ACS Chem. Biol..

[13] Paige J.S., Wu K.Y., Jaffrey S.R. (2011). RNA mimics of green fluorescent protein. Science.

[14] Zimyanin V.L., Belaya K., Pecreaux J., Gilchrist M.J., Clark A., Davis I., St Johnston D. (2008). In vivo imaging of oskar mRNA transport reveals the mechanism of posterior localization. Cell.

[15] Bakshi S., Siryaporn A., Goulian M., Weisshaar J.C. (2012). Superresolution imaging of ribosomes and RNA polymerase in live Escherichia coli cells. Mol. Microbiol..

[16] Sanamrad A., Persson F., Lundius E.G., Fange D., Gynna A.H., Elf J. (2014). Single-particle tracking reveals that free ribosomal subunits are not excluded from the Escherichia coli nucleoid. Proc. Natl. Acad. Sci. U.S.A..

[17] McIntosh B., Ramachandiran V., Kramer G., Hardesty B. (2000). Initiation of protein synthesis with fluorophore-Met-tRNA(f) and the involvement of IF-2. Biochimie.

[18] Schleich H.G., Wintermeyer W., Zachau H.G. (1978). Replacement of wybutine by hydrazines and its effect on the active conformation of yeast tRNAPhe. Nucleic Acids Res..

[19] Blanchard S.C., Gonzalez R.L., Kim H.D., Chu S., Puglisi J.D. (2004). tRNA selection and kinetic proofreading in translation. Nat. Struct. Mol. Biol..

[20] Blanchard S.C., Kim H.D., Gonzalez R.L., Puglisi J.D., Chu S. (2004). tRNA dynamics on the ribosome during translation. Proc. Natl. Acad. Sci. U.S.A..

[21] Fei J., Kosuri P., MacDougall D.D., Gonzalez R.L. (2008). Coupling of ribosomal L1 stalk and tRNA dynamics during translation elongation. Mol. Cell.

[22] Pan D., Qin H., Cooperman B.S. (2009). Synthesis and functional activity of tRNAs labeled with fluorescent hydrazides in the D-loop. RNA.

[23] Wintermeyer W., Zachau H.G. (1979). Fluorescent derivatives of yeast tRNAPhe. Eur. J. Biochem..

[24] Rodnina M.V., Fricke R., Wintermeyer W. (1994). Transient conformational states of aminoacyl-tRNA during ribosome binding catalyzed by elongation factor Tu. Biochemistry.

[25] Kothe U., Rodnina M.V. (2007). Codon reading by tRNAAla with modified uridine in the wobble position. Mol. Cell.

[26] Betteridge T., Liu H., Gamper H., Kirillov S., Cooperman B.S., Hou Y.M. (2007). Fluorescent labeling of tRNAs for dynamics experiments. RNA.

[27] Kaur J., Raj M., Cooperman B.S. (2011). Fluorescent labeling of tRNA dihydrouridine residues: Mechanism and distribution. RNA.

[28] Lee T.H., Blanchard S.C., Kim H.D., Puglisi J.D., Chu S. (2007). The role of fluctuations in tRNA selection by the ribosome. Proc. Natl. Acad. Sci. U.S.A..

[29] Fei J., Bronson J.E., Hofman J.M., Srinivas R.L., Wiggins C.H., Gonzalez R.L. (2009). Allosteric collaboration between elongation factor G and the ribosomal L1 stalk directs tRNA movements during translation. Proc. Natl. Acad. Sci. U.S.A..

[30] Chen J., Petrov A., Johansson M., Tsai A., O'Leary S.E., Puglisi J.D. (2014). Dynamic pathways of -1 translational frameshifting. Nature.

[31] Uemura S., Aitken C.E., Korlach J., Flusberg B.A., Turner S.W., Puglisi J.D. (2010). Real-time tRNA transit on single translating ribosomes at codon resolution. Nature.

[32] Barhoom S., Kaur J., Cooperman B.S., Smorodinsky N.I., Smilansky Z., Ehrlich M., Elroy-Stein O. (2011). Quantitative single cell monitoring of protein synthesis at subcellular resolution using fluorescently labeled tRNA. Nucleic Acids Res..

[33] Barhoom S., Farrell I., Shai B., Dahary D., Cooperman B.S., Smilansky Z., Elroy-Stein O., Ehrlich M. (2013). Dicodon monitoring of protein synthesis (DiCoMPS) reveals levels of synthesis of a viral protein in single cells. Nucleic Acids Res..

[34] Crawford R., Torella J.P., Aigrain L., Plochowietz A., Gryte K., Uphoff S., Kapanidis A.N. (2013). Long-lived intracellular single-molecule fluorescence using electroporated molecules. Biophys. J..

[35] Manley S., Gillette J.M., Patterson G.H., Shroff H., Hess H.F., Betzig E., Lippincott-Schwartz J. (2008). High-density mapping of single-molecule trajectories with photoactivated localization microscopy. Nat. Methods.

[36] English B.P., Hauryliuk V., Sanamrad A., Tankov S., Dekker N.H., Elf J. (2011). Single-molecule investigations of the stringent response machinery in living bacterial cells. Proc. Natl. Acad. Sci. U.S.A..

[37] Uphoff S., Reyes-Lamothe R., Garza de Leon F., Sherratt D.J., Kapanidis A.N. (2013). Single-molecule DNA repair in live bacteria. Proc. Natl. Acad. Sci. U.S.A..

[38] Kaur T., Mukherjea D., Sheehan K., Jajoo S., Rybak L.P., Ramkumar V. (2011). Short interfering RNA against STAT1 attenuates cisplatin-induced ototoxicity in the rat by suppressing inflammation. Cell Death Dis..

[39] Plochowietz A., El-Sagheer A.H., Brown T., Kapanidis A.N. (2016). Stable End-Sealed DNA as Robust Nano-rulers for In Vivo Single-Molecule Fluorescence. Chem. Sci..

[40] Tokunaga M., Imamoto N., Sakata-Sogawa K. (2008). Highly inclined thin illumination enables clear single-molecule imaging in cells. Nat. Methods.

[41] Young J.W., Locke J.C., Altinok A., Rosenfeld N., Bacarian T., Swain P.S., Mjolsness E., Elowitz M.B. (2012). Measuring single-cell gene expression dynamics in bacteria using fluorescence time-lapse microscopy. Nat. Protoc..

[42] Holden S.J., Uphoff S., Hohlbein J., Yadin D., Le Reste L., Britton O.J., Kapanidis A.N. (2010). Defining the limits of single-molecule FRET resolution in TIRF microscopy. Biophys. J..

[43] Crocker J.C., Grier D.G. (1996). Methods of digital video microscopy for colloidal studies. J. Colloid Interface Sci..

[44] Michalet X., Berglund A.J. (2012). Optimal diffusion coefficient estimation in single-particle tracking. Phys. Rev. E Stat. Nonlin. Soft Matter Phys..

[45] Sliusarenko O., Heinritz J., Emonet T., Jacobs-Wagner C. (2011). High-throughput, subpixel precision analysis of bacterial morphogenesis and intracellular spatio-temporal dynamics. Mol. Microbiol..

[46] Plochowietz A., Crawford R., Kapanidis A.N. (2014). Characterization of organic fluorophores for in vivo FRET studies based on electroporated molecules. Phys. Chem. Chem. Phys..

[47] Sustarsic M., Plochowietz A., Aigrain L., Yuzenkova Y., Zenkin N., Kapanidis A. (2014). Optimized delivery of fluorescently labeled proteins in live bacteria using electroporation. Histochem. Cell Biol..

[48] Aigrain L., Sustarsic M., Crawford R., Plochowietz A., Kapanidis A.N. (2015). Internalization and observation of fluorescent biomolecules in living microorganisms via electroporation. J. Vis. Exp..

[49] Thompson R.E., Larson D.R., Webb W.W. (2002). Precise nanometer localization analysis for individual fluorescent probes. Biophys. J..

[50] Dennis P.P., Bremer H. (1974). Differential rate of ribosomal protein synthesis in Escherichia coli B/r. J. Mol. Biol..

[51] Parker J. (1989). Errors and alternatives in reading the universal genetic code. Microbiol. Rev..

[52] Stracy M., Lesterlin C., Garza de Leon F., Uphoff S., Zawadzki P., Kapanidis A.N. (2015). Live-cell superresolution microscopy reveals the organization of RNA polymerase in the bacterial nucleoid. Proc. Natl. Acad. Sci. U.S.A..

[53] Werner A. (2011). Predicting translational diffusion of evolutionary conserved RNA structures by the nucleotide number. Nucleic Acids Res..

[54] Shi H., Moore P.B. (2000). The crystal structure of yeast phenylalanine tRNA at 1.93 A resolution: a classic structure revisited. RNA.

[55] Ando T., Skolnick J. (2010). Crowding and hydrodynamic interactions likely dominate in vivo macromolecular motion. Proc. Natl. Acad. Sci. U.S.A..

[56] Bilgin N., Ehrenberg M., Ebel C., Zaccai G., Sayers Z., Koch M.H., Svergun D.I., Barberato C., Volkov V., Nissen P. (1998). Solution structure of the ternary complex between aminoacyl-tRNA, elongation factor Tu, and guanosine triphosphate. Biochemistry.

[57] Dunkle J.A., Xiong L., Mankin A.S., Cate J.H. (2010). Structures of the Escherichia coli ribosome with antibiotics bound near the peptidyl transferase center explain spectra of drug action. Proc. Natl. Acad. Sci. U.S.A..

[58] Aubin J.E. (1979). Autofluorescence of viable cultured mammalian cells. J. Histochem. Cytochem..

[59] Tynan C.J., Clarke D.T., Coles B.C., Rolfe D.J., Martin-Fernandez M.L., Webb S.E. (2012). Multicolour single molecule imaging in cells with near infra-red dyes. PLoS One.

[60] Potts R.O., Ford N.C., Fournier M.J. (1981). Changes in the solution structure of yeast phenylalanine transfer ribonucleic acid associated with aminoacylation and magnesium binding. Biochemistry.

[61] Antosiewicz J., Porschke D. (1989). Effect of aminoacylation on tRNA conformation. Eur. Biophys. J..

[62] Nenninger A., Mastroianni G., Mullineaux C.W. (2010). Size dependence of protein diffusion in the cytoplasm of Escherichia coli. J. Bacteriol..

[63] Elf J., Li G.W., Xie X.S. (2007). Probing transcription factor dynamics at the single-molecule level in a living cell. Science.

[64] Gouy M., Grantham R. (1980). Polypeptide elongation and tRNA cycling in Escherichia coli: a dynamic approach. FEBS Lett..

[65] Negrutskii B.S., Deutscher M.P. (1991). Channeling of aminoacyl-tRNA for protein synthesis in vivo. Proc. Natl. Acad. Sci. U.S.A..

[66] Mirande M. (2010). Processivity of translation in the eukaryote cell: role of aminoacyl-tRNA synthetases. FEBS Lett..

[67] Defeu Soufo H.J., Reimold C., Linne U., Knust T., Gescher J., Graumann P.L. (2010). Bacterial translation elongation factor EF-Tu interacts and colocalizes with actin-like MreB protein. Proc. Natl. Acad. Sci. U.S.A..

[68] Klumpp S., Scott M., Pedersen S., Hwa T. (2013). Molecular crowding limits translation and cell growth. Proc. Natl. Acad. Sci. U.S.A..

[69] Dong H., Nilsson L., Kurland C.G. (1996). Co-variation of tRNA abundance and codon usage in Escherichia coli at different growth rates. J. Mol. Biol..

[70] Mackie G.A. (2013). RNase E: at the interface of bacterial RNA processing and decay. Nat. Rev. Microbiol..

[71] Wang W., Li G.W., Chen C., Xie X.S., Zhuang X. (2011). Chromosome organization by a nucleoid-associated protein in live bacteria. Science.

[72] Siibak T., Peril L., Dönhöfer A., Tats A., Remm M., Wilson D.N., Tenson T., Remme J. (2011). Antibiotic-induced ribosomal assembly defects result from changes in the synthesis of ribosomal proteins. Mol. Microbiol..

